# Humic acid application improves soil quality and wheat yield in saline-alkali soils

**DOI:** 10.3389/fpls.2026.1807251

**Published:** 2026-03-24

**Authors:** Kailong Zhang, Wenhao Feng, Min Gong, Xiaolong Bai, Jiashen Song, Mengmeng Chen, Xiaodong Ding, Huaizhi Zhang

**Affiliations:** 1 State Key Laboratory of Efficient Utilization of Arable Land in China (the Institute of Agricultural Resources and Regional Planning), Chinese Academy of Agricultural Sciences, Beijing, China; 2 College of Resources and Environment, Qingdao Agricultural University, Qingdao, China; 3 Institute of Environment and Sustainable Development in Agriculture, Chinese Academy of Agricultural Sciences, Beijing, China

**Keywords:** humic acid, root growth, salinization, soil microorganisms, soil quality

## Abstract

**Aims:**

Humic acid (HA), a soil conditioner, is crucial in saline-alkali soil remediation; however, its effects on saline-alkali soil quality, crop yield, and regulatory mechanisms remain unclear.

**Methods:**

A 2-year field experiment was conducted, and data from one experimental year were used in this study to explore the effects of different HA application rates (control: 0 Mg ha^-1^; HA3: 3 Mg ha^-1^; HA7.5: 7.5 Mg ha^-1^; HA15: 15 Mg ha^-1^) on soil microorganisms, soil quality, and wheat growth.

**Results:**

Compared with the control, HA treatment significantly increased soil available potassium (22–45%), available phosphorus (41–53%), and soil organic matter (15–20%), and reduced soil pH (0.1–0.17 units), electrical conductivity (8–27%), chloride ions (3–45%), and sulfate ions (7–34%) in the 0–40 cm soil layer at *p* < 0.05, which may be associated with the significantly increased abundances of *Ascomycota* in the rhizosphere soil. HA treatment improved the soil quality index by 22–60% (0–20 cm) and 7–53% (20–40 cm), compared with the control (All *p* < 0.05). Additionally, the biomass, length, and volume of wheat roots increased by 26–43%, 19–32%, and 18–26%, respectively, which may be attributed to the increase in soil AP content and decrease in pH. These changes may have contributed to the increased in the plant growth index by 15–27%. Regression analysis further confirmed that wheat yield was positively correlated with soil quality and plant growth index.

**Conclusions:**

These results indicate that HA application may contribute to higher wheat yield by improving soil quality and crop growth, with the most notable effects at HA7.5 and HA15 levels. Based on cost-effectiveness, 7.5 Mg ha^-1^ (HA7.5) is recommended as the optimal HA application rate for improving saline-alkali soil quality and wheat productivity.

## Introduction

Soil salinization considerably reduces land productivity and threatens food security (Hopmans et al., 2021). Globally, salinization affects 20% of the arable land and 33% of the irrigated areas (Shrivastav and Kumar, 2015). China has the third-largest saline-alkali soil area in the world, with a total area of approximately 36 million hectares, widely distributed across 17 provinces and regions in the northwest, north, northeast, and coastal zones (Mao et al., 2016). As one of the most important grain-producing regions in China, the North China Plain covers a total area of 300,000 square kilometers. Although accounting for less than 20% of China’s cultivated land, this region supports 25% of the national population and contributes nearly 30% of the national grain output, thus playing an irreplaceable role in ensuring national food and ecological security (Liu et al., 2010). However, soil salinization has become one of the key factors restricting agricultural production in these major grain-producing areas. According to current demographic projections, the global population is expected to reach 9.7 billion by 2050, which will likely result in a 70% increase in food demand (Qiao et al., 2022). Soil salinization inhibits plant growth by reducing the capacity of the soil to retain both moisture and nutrients, and weakening its effectiveness in facilitating cation exchange. Therefore, investigating effective measures to ameliorate saline-alkali soils is of considerable importance for sustainable agricultural development.

Adopting organic fertilizers is recognized as an excellent environmentally friendly strategy, as it not only prevents the deterioration of soil quality but also sustains the long-term sustainable development of agricultural production systems (Elshayb et al., 2022; Shao et al., 2024). Organic fertilizers can enhance soil fertility and provide essential nutrients for crop growth (Shakoor et al., 2021). Humic acid, an important type of organic fertilizer, is a complex natural organic compound containing carboxyl, phenol, and hydroxyl groups; it is widely distributed in soils and aquatic ecosystems (Zhang et al., 2018). Humic acid (HA) application can enhance soil fertility, increase fertilizer efficiency, and improve crop growth and stress resistance (Yang and Antonietti, 2020). By optimizing soil architecture and modulating microbial dynamics, humic acid considerably enhances the fertility of soils affected by salt stress (Ouni et al., 2014). Humic acid can increase the soil nutrient content and improve soil microbial diversity, which is beneficial for crop growth and yield (Debska et al., 2016; Li et al., 2019). Previous studies on the application of humic acid for saline-alkali soil remediation have primarily focused on mildly affected areas (Liu et al., 2023; Sun et al., 2021), whereas its efficacy and underlying mechanisms in moderate to severely saline-alkaline soils remain poorly understood and require further investigation.

The root system is crucial for soil resource acquisition and nutrient absorption and supports aboveground plant growth, ultimately determining crop yield and productivity (Li et al., 2023). External environmental factors such as temperature, water availability, and nutrient availability exert particularly significant impacts on highly sensitive root systems (Karlova et al., 2021; Maurel and Nacry, 2020; Poirier et al., 2018). Applying humic acid to the soil can optimize environmental properties including nutrient status, measured pH value, and intrinsic biological activity, thereby ultimately achieving the effect of promoting root growth (Feng et al., 2010). The application of humic acid can increase root fresh weight, root volume, root surface area, and root length (Zandonadi et al., 2019). Similarly, de Castro et al. (2021) revealed that the promoting effect of humic acid on root growth was concentration dependent, primarily by enhancing cell division in the meristematic zone and simultaneously promoting cell elongation in the elongation zone, collectively contributing to an increase in root biomass. However, the effects of different HA dosages of humic acid on root traits under saline-alkali soil conditions and their roles in increasing crop yield remain unclear.

Considering their role in decomposing organic matter and cycling nutrients, soil microorganisms are crucial for sustaining agroecosystem functions and crop yields (Hartmann and Six, 2023; Luo et al., 2018). Fertilization exerts a substantial impact on both the dynamics of soil nutrients and plant growth, primarily by altering the microbial community structure and the abundance of keystone species (Guo et al., 2020). The application of humic acid results in a two-fold increase in soil organic matter content, thereby providing additional carbon sources and energy that facilitate the growth and reproduction of soil microorganisms. Additionally, humic acid enhances the availability of soil nutrients, such as the chelating effect on nitrogen, and indirectly influences microbial metabolic activity (Dehsheikh et al., 2020). Specifically, Maji et al. (2017) demonstrated that humic acid application increased the Shannon and Margalef indices of both bacteria and fungi, and promoted plant growth and soil health by modifying the soil environment. Furthermore, humic acid-microbial combinations alter the composition of soil microorganisms, increase the bacteria/fungi ratio, and modulate specific bacterial and fungal populations, ultimately improving ecosystem functionality and plant nutrient acquisition (Cozzolino et al., 2021). Additionally, soil quality plays a critical role in two key agricultural processes that facilitate the recycling of nutrients while supporting the long-term sustainability of agricultural production systems (He et al., 2022; Qiao et al., 2022). To date, numerous studies have used the soil quality index (SQI) as a tool for evaluating soil quality. The application of organic fertilizers has been confirmed by studies to not only improve soil nutrient status but also effectively enhance the SQI (Ling et al., 2024; He et al., 2022). However, studies investigating the influence of humic acid on the soil quality index (SQI) are limited.

However, our understanding is still limited of how humic acid affects crop yield through root development, soil quality, and microbial interactions, particularly in moderately and severely saline-alkali soils, where studies remain relatively scarce. Therefore, this study aimed to: (1) evaluate the effects of different gradients of humic acid (HA) on root growth and soil quality; (2) identify the variation patterns of key soil microbial taxa under different HA application gradients; and (3) reveal how different HA gradients regulate wheat yield improvement through their interactions with wheat root growth, soil quality, and soil microorganisms, and elucidate the underlying mechanisms. This analysis was based on a 2-year field experiment conducted in moderate-to-severe saline-alkaline soils in Shandong Province, China, with data from a single experimental year selected for the present study. We hypothesized that: (1) HA can facilitate wheat root development and ameliorate soil quality by increasing the abundance of keystone microbial taxa, which consequently contributes to increased wheat yield, and (2) in saline-alkaline soil environments, the influence of HA on soil microbial communities and wheat yield is strongly associated with the quantity of HA applied, with a specific optimal dosage identified.

## Materials and methods

### Study site description

Field experiments were conducted at the Dezhou Experimental Station of the Chinese Academy of Agricultural Sciences (CAAS), located in Haowangzhuang Town, Dezhou City, Shandong Province (37.43° N, 116.36° E). The experimental site was within a climate zone classified as warm-temperate continental monsoon. In this zone, the annual mean rainfall is 504.9 mm, and the mean annual temperature is 14.9 °C. The soil at the experimental site was categorized as fluvoaquic. The basic soil properties of the test site are listed in **Table 1**. All soil properties were determined using the analytical methods described by Bao (2000).

**Table 1:** Soil properties of the sampling area in the 0–40 cm soil layer.

Soil layer	pH	SOM (g·kg^-1^)	TN (g·kg^-1^)	TP (g·kg^-1^)	TK (g·kg^-1^)	AHN (mg·kg^-1^)	AP (mg·kg^-1^)	AK (mg·kg^-1^)
0-20cm	8.16	1.13	0.10	0.99	16.52	40.00	12.00	126.00
20-40cm	8.25	0.36	0.02	0.45	14.51	14.53	2.27	47.73

### Experimental design

A completely randomized block design was employed in a 2-year field experiment, with data from one representative year selected for the present study. Each treatment was replicated thrice (n = 3), and the area of each plot was set to 90 m^2^ (15 × 6 m). Three rates of HA were applied in the experiment: 3 (HA3), 7.5 (HA7.5), and 15 (HA15) Mg ha^-1^, with a control treatment (CK) receiving no humic acid. The experiment comprised four treatments: no humic acid application (CK), humic acid application at 3, 7.5, and 15 Mg ha^-1^. Commercial humic acid (HA) from Xiubang Ecological Technology Co., Ltd., Henan, China was used, and its main properties were as follows: pH 3.55, total nitrogen (TN) 7.8 g kg^−1^, organic matter content 417 g kg^−1^, total phosphorus (TP) 1.2 g kg^−1^, and total potassium (TK) 1.8 g kg^−1^.

The seeding rate was 450 kg ha^-1^ with a row spacing of 15 cm. All plots were treated with an organic-inorganic compound fertilizer application based on the rates of local farmers: 120 kg N ha^-1^, 90 kg P ha^-1^, and 30 kg K ha^-1^, with an additional topdressing of 67.5 kg N ha^-1^ on March 25, 2025. Prior to sowing, humic acid (HA) and compound chemical fertilizers were first applied to the soil surface and then uniformly incorporated into the upper 20 cm soil layer using a rotary tiller. To promote seed germination, 30 mm of irrigation water was applied to all the plots immediately after sowing. No pesticides or herbicides were used during the experimental period. All other agronomic management practices were consistent with conventional local agricultural measures.

### Plant material sampling and analytical examination

#### Evaluation of nutrient status in plant tissues and yield-related indicators

Plant samples for nutrient content analysis were collected on June 6, 2025, during the grain maturity stage. Two sample sets were randomly collected from each experimental plot, each containing wheat plants grown continuously for more than 1 m. To determine aboveground biomass, fresh samples of aboveground plant parts were oven-dried at 65 °C until they reached a constant weight. A subsample was collected from the dried material, which was first ground into a fine powder and subsequently passed through a 0.15-mm sieve. TN was measured using a Kjeldahl analyzer (Analytik Jena, Germany). Wheat grain yield was precisely quantified by harvesting, air-drying, and threshing samples from a 1-m² area per plot. Yield components (spike number, grains per spike, and 1000-grain weight) were determined following established methods.

#### Sampling and analysis of wheat roots

On May 12, 2025 (wheat filling stage), root samples were collected from the 0–20 cm soil layer using a single-root auger (depth: 200 mm, diameter: 70 mm). Root samples were placed in nylon bags and thoroughly rinsed with tap water to carefully separate them from the soil particles that had adhered to their surfaces. Detailed information on the sampling and analysis of wheat roots is provided in the Supplementary File, Text S2.

### Collection and analytical examination of soil samples

#### Physicochemical properties of soil samples

On June 6, 2025, during the grain maturity period, a five-point sampling method was used to collect soil samples at depths of 0–20 cm and 20–40 cm. Each soil sample was split into two parts: one portion of fresh soil was stored at –20 °C for soil biological analysis, and the other was air-dried, sieved through a 2-mm mesh, and manually freed of plant roots and stones before physicochemical analysis.

#### Soil microbial DNA isolation and sequencing

DNA extraction was performed using the OMEGA Soil DNA Kit (Cat. No. M5635-02; Omega Bio-Tek, Norcross, GA, USA), The extracted DNA was stored at -80 °C for subsequent analyses. For the quantification and quality assessment of the extracted DNA, two methods were sequentially employed: first, a NanDrop NC2000 spectrophotometer (Thermo Fisher Scientific, Waltham, MA, USA), followed by agarose gel electrophoresis. Comprehensive details regarding the high-throughput sequencing and sequence analysis are provided in the Supplementary File Text S3.

#### Calculations plant growth index and soil quality index

Radar charts were constructed based on all soil indicators, and the soil quality index (SQI) was further determined by comparing the areas of these radar charts (Kuzyakov et al., 2020).

(4) $$SQI_{-area}=0.5\cdot \sum_{i}^{n}SL_{i}^{2}\cdot \sin\left(\frac{2\pi }{n}\right)$$

where *n* represents the quantity of soil indicators used to compute the SQI, and *“SL_i_”* refers to the linear score corresponding to the *i*^th^ soil indicator. Detailed information regarding the calculation method of the linear score is provided in the **Supplementary File Text S4**.

Following the same underlying principle and methodology used in determining the SQI, the plant growth index (PGI) was obtained using Equation 4. This index comprehensively assesses the growth status of plants (Nayab et al., 2022).

The SQI calculated in this study includes water-soluble ions, pH, EC, total nitrogen (TN), total phosphorus (TP), total potassium (TK), alkali-hydrolyzable nitrogen (AN), available phosphorus (AP), and available potassium (AK); The PGI calculated in this study includes the relative responses of root traits, as well as nitrogen (N), phosphorus (P), and potassium (K) contents in roots and shoots.

To ensure clarity and conciseness, detailed protocols are provided in the supplementary materials: Supplementary Text S2: Soil physicochemical property determination methods; Supplementary Text S3: Soil microbial DNA extraction, sequencing, and bioinformatic analysis procedures; Supplementary Text S4: Calculation methods for the soil quality index (SQI) and plant growth index (PGI).

### Statistical analytical procedures

To verify the normality of data distribution, the Shapiro-Wilk test was performed prior to all additional analyses. A one-way analysis of variance (ANOVA) was used to assess the influence of humic acid treatments on wheat yield, yield components, straw nitrogen uptake, and root traits, soil physicochemical properties, microbial α-diversity indices, and the dominant taxa of bacteria and fungi. Regression analysis was employed to examine the correlation between wheat yield and the plant growth index (PGI) as well as the soil quality index (SQI). Non-metric multidimensional scaling (NMDS) was applied to explore changes in bacterial and fungal communities using the “vegan” package. The Mantel test was used to investigate the effects of three factors (plant growth indicators, soil properties, and key microbial taxa) on wheat yield components using the “linkET” package. Random forest model was employed using the “rfPermute” package in R 4.1.3 to identify the key factors that affect PGI and SQI.

## Results

### Wheat grain yield and related variables of wheat

**Figure 1:**
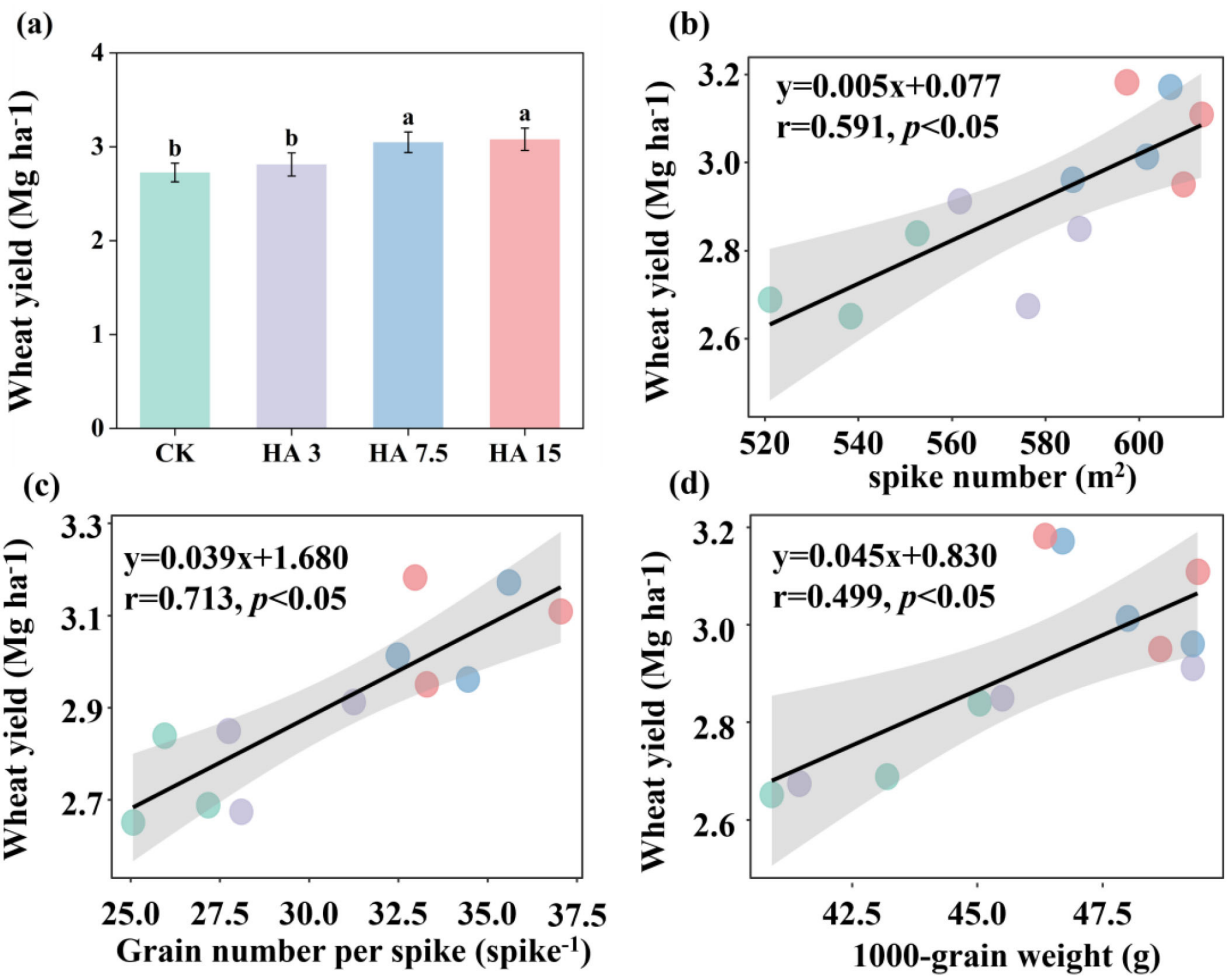
Effect of humic acid (HA) application on wheat yield **(a)**, and the correlations between wheat yield and spike number **(b)**, grains per spike **(c)**, and 1000-grain weight **(d)**. Data are presented as mean ± standard deviation (*n* = 3), with significant differences among treatments at the 0.05 level marked with different letters. Black lines represent regression lines, and shaded areas indicate the 95% confidence intervals. HA treatments included CK (0 Mg ha^-1^), HA 3 (3 Mg ha^-1^), HA 7.5 (7.5 Mg ha^-1^), and HA 15 (15 Mg ha^-1^).

Compared to that of CK, HA application increased wheat yield by 3–13%, with the two highest application rates (HA 7.5 and HA 15) resulting in significant yield increases (*p* < 0.05, **Figure 1**). The highest wheat yield was obtained in HA15 (3.08 Mg ha^-1^), followed by HA7.5 (3.04 Mg ha^-1^) and HA3 (2.81 Mg ha^-1^), whereas the yield in CK was the lowest (2.72 Mg ha^-1^) (**Figure 1**). Linear regression analysis revealed a positive relationship between wheat yield and the number of grains per spike (*r* = 0.71, *p* < 0.05), spike number (*r* = 0.59, *p* < 0.05), and 1000-grain weight (*r* = 0.49, *p* < 0.05) (**Figure 1**, **Table 2**).

**Table 2:** Effect of humic acid (HA) application on wheat yield components. HA treatments included CK (0 Mg ha^−1^), HA 3 (3 Mg ha^−1^), HA 7.5 (7.5 Mg ha^−1^), and HA 15 (15 Mg ha^−1^).

Treatments	Spike number (m^-2^)	Grain number (spike^-1^)	1000-grain weight (g)
CK	161.21 ± 4.73c	26.06 ± 1.06b	4.31 ± 0.21a
HA 3	172.52 ± 3.85b	29.03 ± 1.93b	4.54 ± 0.39a
HA 7.5	179.42 ± 1.46ab	34.18 ± 1.58a	4.80 ± 0.13a
HA 15	182.02 ± 2.49a	34.44 ± 2.26a	4.81 ± 0.13a
One-way ANOVA			
Treatment	<0.001	<0.001	0.100

If data are followed by different letters, it indicates that there are significant differences among different treatments at the 0.05 level. Values are presented as means ± standard deviation (*n* = 3).

### Plant growth index and root morphological traits

**Figure 2:**
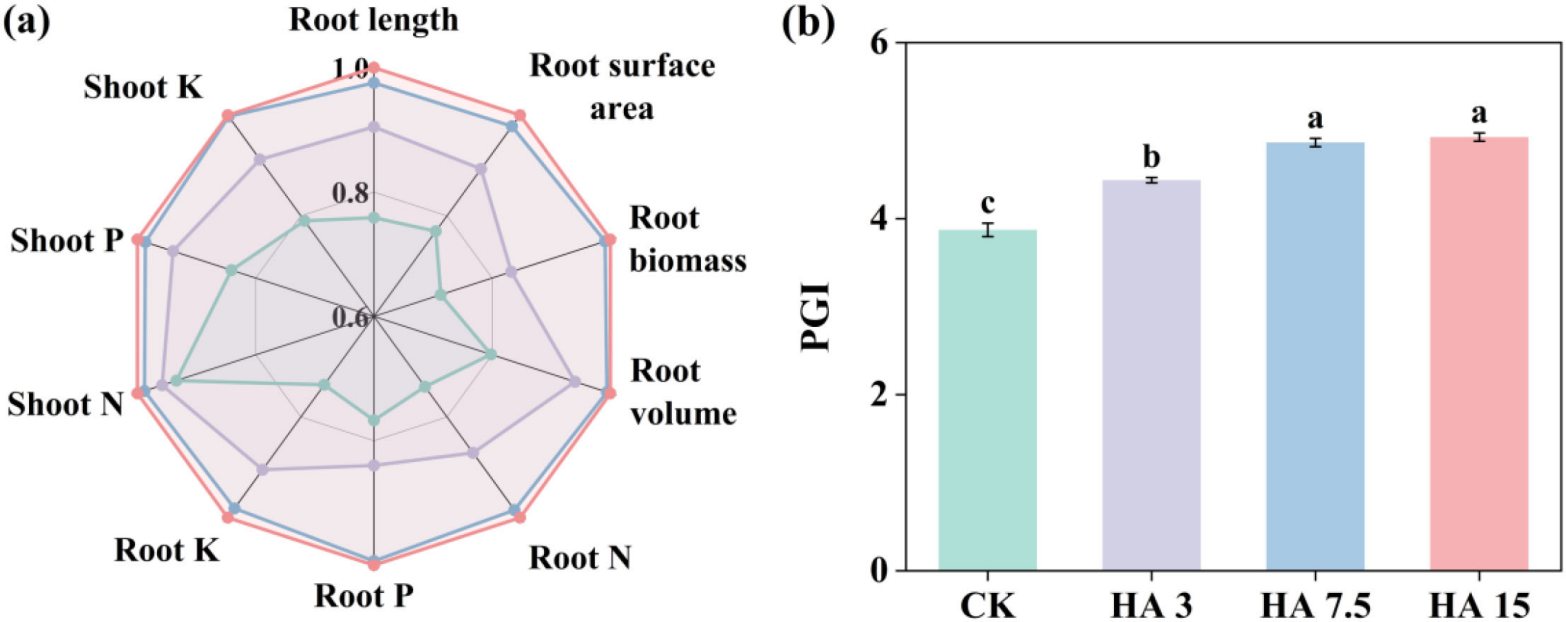
Radar chart: relative responses of root traits, root and shoot N, P, and K uptake to different application rates of humic acid (HA) **(a)**, and the plant growth index (PGI) area **(b)**. In panel **(a)**, the relative response values were normalized to the control treatment (CK, 0 Mg ha^-1^) and scaled to a range of 0–1. The different colored lines represent: CK (green), HA3 (3 Mg ha^-1^, light purple), HA7.5 (7.5 Mg ha^-1^, blue), and HA15 (15 Mg ha^-1^, red). Data are presented as mean ± standard deviation (*n* = 3). Significant differences among treatments at the 0.05 level are marked with different letters. HA treatments included CK (0 Mg ha^-1^), HA3 (3 Mg ha^-1^), HA7.5 (7.5 Mg ha^-1^), and HA15 (15 Mg ha^-1^).

HA7.5 and HA15 notably increased root length, surface area, biomass, and volume by 28% and 32%, 27% and 30%, 39% and 40%, and 24% and 25%, respectively, compared with those of CK (**Figure 2**, **Supplementary Table 1**). HA application improved the uptake of root nitrogen (N), P, and root K by 18–35%, 10%–30%, and 23–36%, respectively. Moreover, the uptake of shoot N, P, and K under HA treatment increased by 3–7%, 12–19%, and 15–27%, respectively. Overall, plant growth index (PGI) in the HA-treated groups was 15–27% higher than that in the CK group (*p* < 0.05, **Figure 2**).

### Soil quality index and related indices

**Figure 3:**
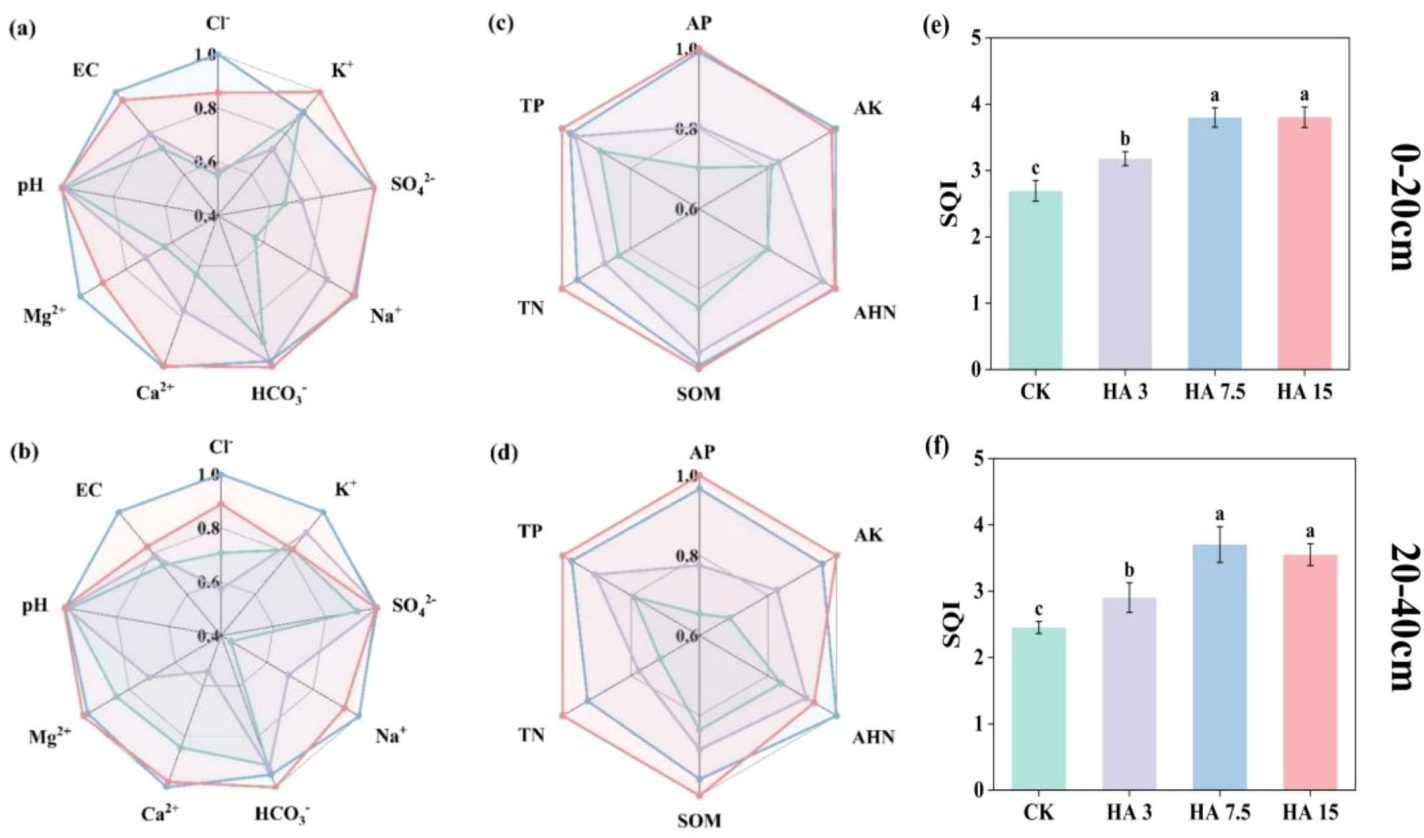
Radar charts showing the relative responses of soil fertility indicators to different humic acid (HA) application rates in the 0–20 cm **(a, c)** and 20–40 cm **(b, d)** soil layers, and the corresponding soil quality index (SQI) **(e, f)**. In panels **(a–d)**, the relative response values were normalized to the control treatment (CK, 0 Mg ha^−1^) and scaled to a range of 0–1. The different colored lines represent: CK (green), HA3 (3 Mg ha^−1^, light purple), HA7.5 (7.5 Mg ha^−1^, blue), and HA15 (15 Mg ha^−1^, red). Data are presented as mean ± standard deviation (SD, n=3). Different letters indicate significant differences among treatments at the 0.05 level. Cl^−^, chloride ion; K^+^, potassium ion; SO_4_^2−^, sulfate ion; Na^+^, sodium ion; HCO_3_^-^, bicarbonate ion; Ca^2+^, calcium ion; Mg^2+^, magnesium ion; pH, potential of hydrogen; EC, electrical conductivity; AP, available phosphorus; AK, available potassium; AHN, alkaline hydrolyzable nitrogen; SOM, soil organic matter; TN, total nitrogen; TP, total phosphorus.

In the 0–20 cm layer, HA7.5 and HA15 significantly reduced soil pH by 0.14 and 0.16 units, respectively, and EC by 27% and 24%, respectively, compared with CK. In the 20–40 cm layer, the reductions in pH were 0.13 and 0.17 units, and in EC were 26% and 11%, respectively (*p*< 0.05; **Figures 3a, b**; **Supplementary Table 2**). However, in the 0–20 cm soil layer, HA7.5 and HA15 increased the AHN and TN contents by 24–25% and 14–20%, respectively (*p* < 0.05, **Supplementary Table 2**). HA7.5 and HA15 also increased AP by 41–42% and 48–53%, AK by 22–23% and 39–45%, SOM by 17–18% and 15–20%, and TP by 10–13% and 3–26% in the 0–20 and 20–40 cm soil depths (*p* < 0.05, **Supplementary Table 3**). Additionally, in the 0–20 and 20–40 cm soil layers, HA7.5 and HA15 significantly increased SQI compared to CK by 40–41% and 45–51%, respectively (*p* < 0.05, **Figures 3e, f**).

### Composition and diversity of the microbial community

**Figure 4:**
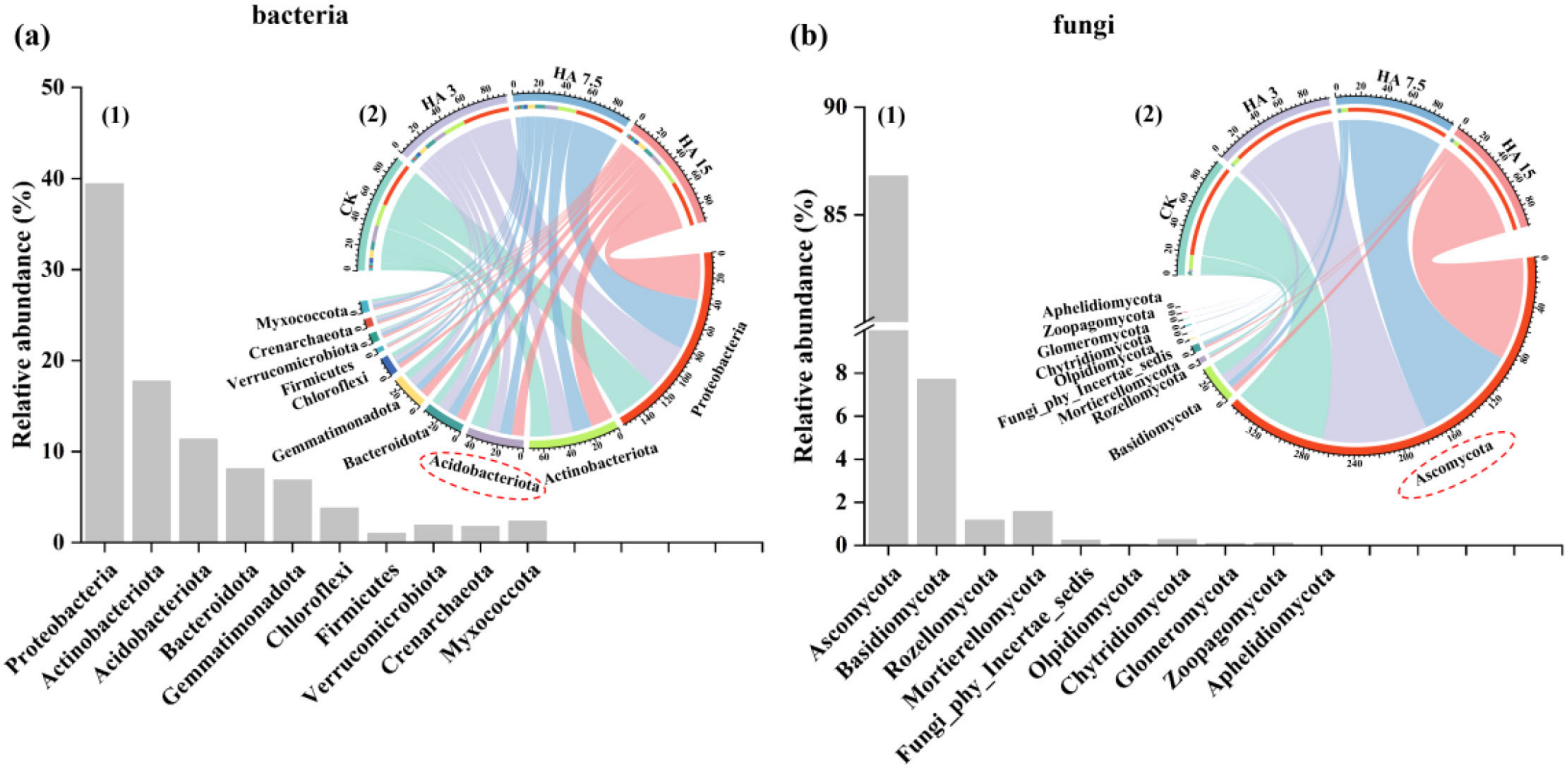
Results of community composition analysis of rhizosphere soil bacteria **(a)** and fungi **(b)**: (1) showing the total relative abundance of the top 10 species at the phylum level; (2) illustrating their distribution under different treatments. The red circle markers represent significant differences among treatments at the 0.05 level. HA treatments included CK (0 Mg ha^−1^), HA 3 (3 Mg ha^−1^), HA 7.5 (7.5 Mg ha^−1^), and HA 15 (15 Mg ha^−1^).

Although humic acid (HA) application did not significantly change the α-diversity of soil bacterial and fungal communities (as indicated by the Chao1 and Shannon indices), it induced notable changes in their community composition (**Supplementary Table 4** and **Supplementary Figure 1**). At the phylum level, the relative abundance of Acidobacteria was significantly enriched in the HA7.5, and HA15 groups, exceeding that in the CK by 13% and 28%, respectively (*p* < 0.05, **Figure 4a**). Regarding the fungal community, the relative abundance of Ascomycota was significantly higher in the HA3, HA7.5, and HA15 groups by 13%, 10%, and 10% respectively, than that in the control treatment (*p* < 0.05, **Figure 4b**).

### Correlations

**Figure 5:**
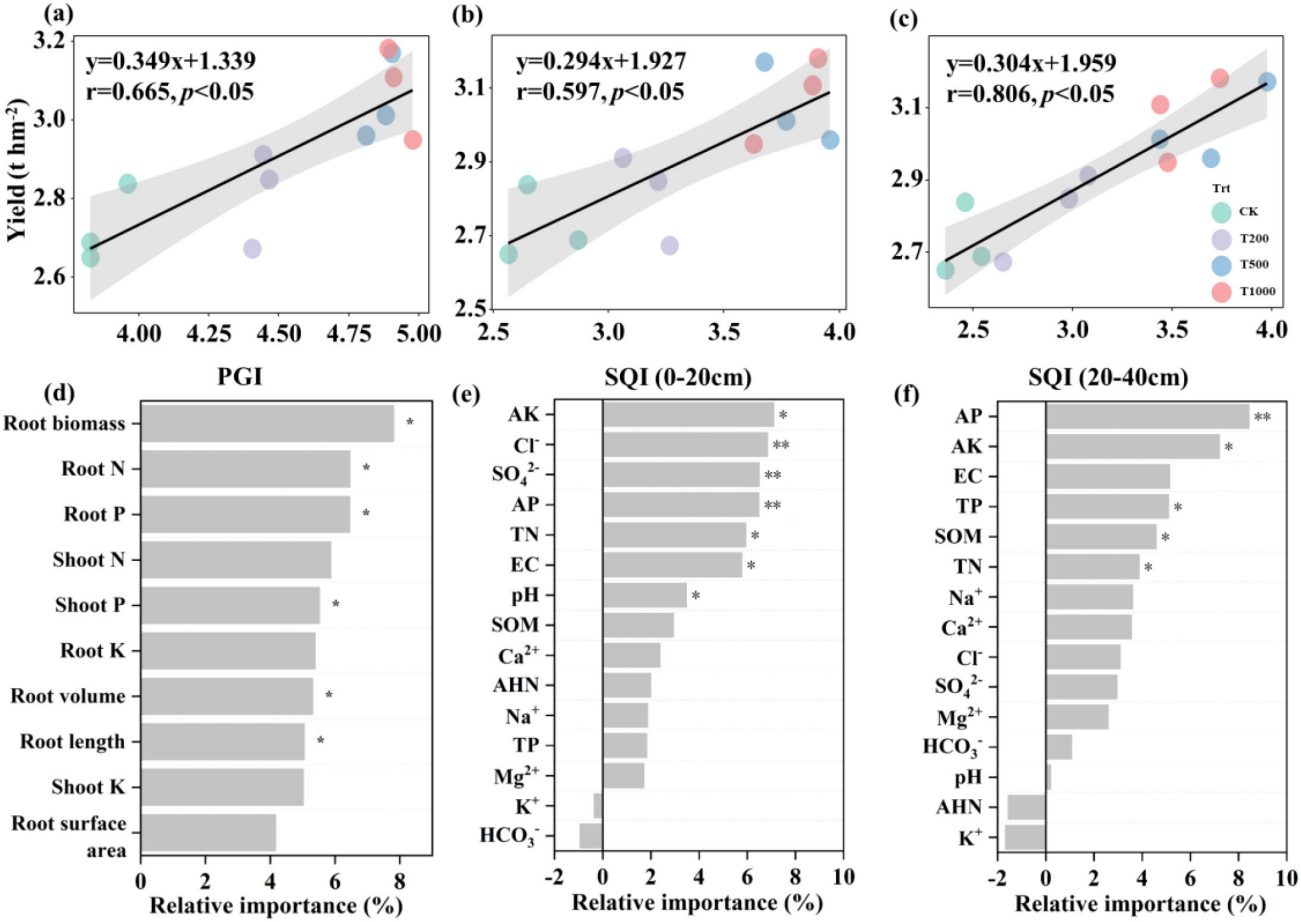
Relationships between wheat yield and plant growth index (PGI) **(a)**, soil quality index (SQI) in 0–20 cm soil layer **(b)**, and SQI in 20–40 cm soil layer **(c)**, as well as random forest models for predicting the key factors of PGI **(d)** and SQI in each soil layer **(e, f)**. Black lines in the figures represent regression lines, and shaded areas indicate the 95% confidence intervals. *p < 0.05, **p < 0.01.

Wheat yield was positively correlated with PGI and SQI at both the 0–20 and 20–40 cm soil depths via linear regression analysis (*p* < 0.05, **Figure 5C**). Random forest models provided further evidence that root biomass was ranked as the primary predictor of PGI, followed by root nitrogen and phosphorus uptake (**Figure 5D**). At the 0–20 cm soil depth, AK was identified as the primary predictor of SQI, followed by Cl^-^, SO_4_^2-^, AP, TN, EC, and pH (**Figure 5E**). At a soil depth of 20–40 cm, AP was the primary predictor of SQI, followed by AK, TP, SOM, and TN (**Figure 5F**).

## Discussion

### Humic acid increases crop yield by regulating root traits and enhancing soil quality

By optimizing yield components (notably spike number, grains per spike, and 1000-grain weight), humic acid (HA) significantly increased wheat yield. Li et al. (2025) reported that HA improves soil structure, increases fertilizer retention capacity, and facilitates the absorption of nutrients such as nitrogen (N) and phosphorus (P) by the roots, thereby increasing grain number and weight through improved nutrient accumulation and biomass synthesis, ultimately increasing crop yields. Their results are consistent with those of the current study. In this study, the plant growth index (PGI) was significantly higher in HA-treated groups (**Figure 2**), and PGI showed a positive correlation with wheat yield (**Figure 5**), further verifying the promotional effect of HA on yield formation. HA treatment also significantly increased wheat root length, surface area, and volume, which is consistent with previous studies indicating that HA can enhance crop nutrient acquisition capacity by promoting root growth (Cozzolino et al., 2021; de Castro et al., 2021; Zandonadi et al., 2019). HA can improve the soil environment in saline-alkaline lands, thereby promoting root development and plant growth, with effects increasing with increasing application rate (Ibrahim et al., 2024; Azeem et al., 2015). However, the present study identified a “moderation effect” in the application HA, with optimal performance observed at a moderate application rate of 7.5 Mg ha^−1^. Excessive HA application can lower the bioavailability of nutrients because the large specific surface area and rich functional groups of HA lead to strong adsorption of soil nutrients, thereby indirectly inhibiting crop growth (Nallanthighal et al., 2024). This phenomenon revealed the specificity of the role of HA in saline-alkaline soils. The increased application rates do not yield better results, but rather the effectiveness of HA is regulated by the balance of the soil-plant system.

Wheat yield and soil SQI exhibit a significant positive linear correlation, corroborating that HA boosts wheat yield through soil quality improvement (**Figure 5**), which is consistent with previous studies (Seyedbagheri, 2010; Zhang et al., 2024). HA optimizes the soil environment through two main pathways. HA regulates soil salt content and pH; notable reductions in both parameters were observed in the HA7.5 and HA15 treatments. This is closely related to the chemical properties of HA. In high-salt and high-pH environments, HA maintains a dispersed state through anti-flocculation, reducing damage to the soil colloid structure caused by salt ions and alleviating soil compaction (Li et al., 2025). Meanwhile, HA preferentially adsorbs Na^+^ through cation bridging, reducing its free concentration to mitigate phytotoxicity and supplementing trace elements by strongly binding Ca^2+^ and Mg^2+^, thereby improving the soil ion balance (Abu-Ria et al., 2024; Wang et al., 2025). The contents of soil nutrients and SOM were significantly increased by the HA treatments (**Figure 3**). HA promotes the secretion of organic acids from plant roots to chelate metal ions (e.g., Ca^2+^, Mg^2+^) in saline−alkali soils, activating precipitated phosphorus and reducing phosphorus fixation (Alsudays et al., 2024). It can also form chelate complexes with cationic metals to further inhibit phosphorus immobilization, and synergize with mineral potassium to boost root organic acid exudation for enhanced phosphorus activation (Al-Taweel and Al-Saadawi, 2021; Elshamly and Nassar, 2023). As an available carbon source, HA stimulates rhizosphere microorganisms to secrete abundant phosphatases, accelerating organic phosphorus mineralization (Yang et al., 2024), and also promotes the growth of beneficial microbes to optimize the rhizosphere environment for phosphorus acquisition (Al-Taweel and Al-Saadawi, 2021). Humic acid (HA) is abundant in negatively charged functional groups, such as carboxyl and phenolic hydroxyl groups, which enhance the soil cation exchange capacity (CEC), increase the adsorption of K^+^, and reduce leaching loss. HA is crucial in facilitating the formation of soil aggregates, optimizing pore structure, accelerating the dissolution and transformation of mineral potassium, and maintaining a stable supply of available potassium (Alsudays et al., 2024; Xu et al., 2024). HA also enhances the absorption capacity of ammonium ions (NH_4_^+^) and nitrate ions (NO_3_^-^) by promoting root growth (increasing root length and root surface area), reducing the leaching loss of available nitrogen, and indirectly maintaining the accumulation of total soil nitrogen (Saidimoradi et al., 2019). Simultaneously, HA provides carbon sources and a suitable microenvironment for microorganisms, and microbial activity can promote the mineralization and immobilization of organic nitrogen, thereby reducing nitrogen loss through gaseous emissions or leaching (Baldi et al., 2010). Notably, changes in indicators, such as PGI, were attributed to HA application influencing wheat roots by regulating soil pH and AP levels (**Supplementary Figure 2**).

The optimal HA application rate identified in the present study was 7.5 Mg ha^-1^. When this optimal rate was exceeded (15 Mg ha^-1^), no further improvements in the soil quality were observed and saturation or reversal of some beneficial effects occurred (**Figure 3**). This could be attributed to the limited capacity of the saline-alkali soil to transform and accommodate exogenous HA; excessive HA may disrupt the nutrient balance in the soil-plant system by over-chelating soil cationic metals (e.g., Ca^2+^, Mg^2+^) and intensifying rhizosphere microbial carbon competition, and even induce abnormal soil physicochemical properties that inhibit nutrient bioavailability (Al-Taweel and Al-Saadawi, 2021; Elshamly and Nassar, 2023; Nallanthighal et al., 2024). This result explains the dose-dependence of HA efficacy in moderate-to-severe saline-alkaline soils, supporting the hypothesis that “an appropriate application rate exists in the saline-alkaline soil-plant system.”

In conclusion, this study confirmed that HA synergistically improves yield components by promoting wheat root growth (enhancing resource acquisition) and improving soil quality (optimizing salt conditions and nutrient availability), ultimately increasing wheat yield. In the tested moderate-to-severe saline-alkaline soils, 7.5 Mg ha^-1^ was the optimal HA application rate, with excessive application potentially exerting negative effects. These results validate the research hypothesis that HA improves yield by regulating root-soil interactions and that its efficacy is dependent on the application rate.

### Response of soil microorganisms to humic acids

Humic acid (HA) application modified the soil microbial communities and increased the abundance of key microbial taxa, which was consistent with our initial hypothesis. HA treatments notably affected fungal communities, but did not significantly affect bacterial communities (**Supplementary Figure 3**). This may be because of two reasons: First, the carbon sources and microenvironments provided by HA may be more compatible with the metabolic characteristics of fungi, potentially conferring a competitive advantage over bacteria in terms of community proliferation and functional performance (Maji et al., 2017). Second, HA-induced reductions in soil pH and improvements in aggregate structure in saline-alkaline soils may promote fungal growth, as fungi typically exhibit broader adaptability to weakly alkaline to neutral pH conditions (Li et al., 2019). In contrast, bacteria are more sensitive to pH fluctuations, and their proliferation may be inhibited by HA treatment. Consistently, HA treatment increased the relative abundances of *Acidobacteriota* and *Ascomycota* in the rhizosphere soil compared with the control (**Figure 4**). This is consistent with the proposed mechanism that HA functions through a synergistic “carbon supply–community regulation–microenvironment optimization–function activation” pathway. This pathway can stimulate the soil microbial activity more precisely and efficiently. HA enhances community abundance and diversity as well as directionally enriches beneficial taxa involved in nutrient transformation and disease suppression. HA enhances microbial metabolic activity, thereby providing a sustained biological driving force for the long-term restoration of saline-alkaline soil ecosystems (da Silva et al., 2021).

**Figure 6:**
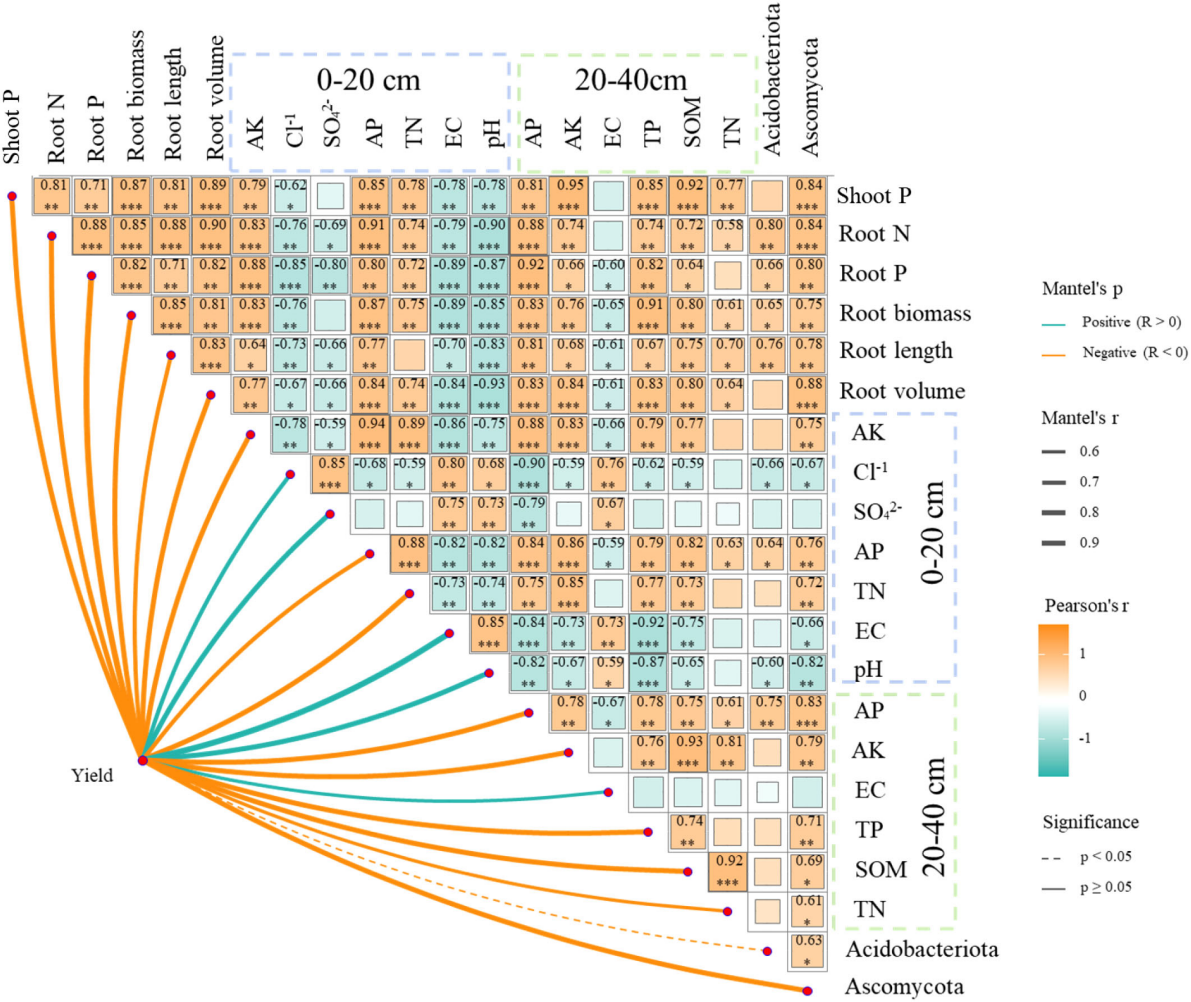
Key factors affecting wheat yield, plant growth index (PGI), and soil quality index (SQI) in soil layers at different depths (0–20 cm, 20–40 cm), as well as yield components of wheat. *p < 0.05, **p < 0.01, ***p < 0.001. Shoot P, plant phosphorus content; Root N, root nitrogen content; Root P, root phosphorus content; Root biomass, root weight; Root length, root length; Root volume, root surface area; AK, available potassium; Cl^−^, chloride ion; SO_4_^2−^, sulfate ion; AP, available phosphorus; TN, total nitrogen; EC, electrical conductivity; pH, potential of hydrogen; SOM, soil organic matter; *Acidobacteriota*, *Acidobacteria*; *Ascomycota*, *Ascomycetes*.

A significant negative association was observed among the abundance of Acidobacteria, soil electrical conductivity (EC), and pH (**Figure 6**), indicating that changes in *Acidobacteriota* abundance may be driven by HA-induced alterations in the soil environment. Both the distribution and functional roles of Acidobacteria are strongly influenced by soil pH levels. Low-pH environments can significantly increase their abundance and activity by enhancing their physiological adaptability and nutrient availability (Song et al., 2025). Additionally, plants can reduce the soil pH through root exudates and transpiration, thereby indirectly promoting the proliferation of *Acidobacteriota*. Additionally, we observed that the improvement in soil quality may be associated with an increase in the abundance of *Ascomycota*. Billah et al. (2019) reported that nitrogen (N), phosphorus (P), and potassium (K) are essential elements for soil microbial growth and reproduction, with changes in their availability closely regulating Ascomycota community structure in saline-alkali soils. As the dominant fungal phylum in HA-amended saline-alkali soils (Liu et al., 2019), Ascomycota influences soil fertility and nutrient cycling by secreting hydrolases to decompose refractory organic matter, mediating N/P transformation, and interacting synergistically with exogenous HA (Liu et al., 2019; Orlova et al., 2023). The overall ecological impact depends on the relative proportions of saprophytic, symbiotic, and parasitic taxa within the community. As they efficiently decompose organic matter and are adaptable to low-nutrient environments, these microorganisms are more likely to become the dominant taxa under nutrient-limited conditions (Ma et al., 2024; Wang et al., 2020). Within the framework of this study, the higher abundance of *Ascomycota* in the rhizosphere soil of the HA-treated plots further confirmed these observations. Notably, increased abundances of both *Acidobacteriota* and *Ascomycota* were associated with the suppression of soil pathogens and were positively correlated with plant growth indicators (**Figure 6**). In summary, the application of HA led to a higher abundance of beneficial microbial taxa associated with organic matter turnover and the suppression of plant diseases, thereby promoting plant growth and crop yield. These findings further refined our initial hypothesis, confirming that HA increases the abundance of key microbial taxa. Our second hypothesis stated that the effects of humic acid (HA) on microbial community structure, activity, and wheat yield are associated with application rates, and these results provide support for this.

However, it should be emphasized that this study was based on a one-year field experiment. A single-year experiment has certain limitations in evaluating the long-term effects and interannual stability of HA amendment in the saline-alkali soil environment, as soil properties, microbial communities, and crop responses usually exhibit obvious interannual variability under field conditions. In future research, long-term and multi-year monitoring experiments will be conducted to further verify the sustained improvement effects of HA on saline-alkali soils and wheat growth, so as to provide more systematic and reliable scientific support for practical application.

## Conclusion

In summary, humic acid (HA) application effectively ameliorated saline-alkaline soil properties and promoted crop growth in short-term experiments. The application of HA at 7.5 Mg ha^−1^ exhibited the optimal comprehensive effects, as it significantly improved soil quality in the 0–40 cm layer, facilitated wheat root development, and ultimately contributed to increased plant growth index (PGI) and wheat yield—with comparable improvement effects to higher HA rates but better cost-effectiveness. These beneficial outcomes may be associated with HA-induced improvements in soil physicochemical properties and regulation of rhizosphere microbial communities.

Notably, this study was based on short-term field experiments, and thus future research should focus on long-term monitoring to verify the sustained effects of HA application on saline-alkaline soil improvement and crop productivity, thereby providing more systematic scientific support for its practical application.

## Data Availability

The original contributions presented in the study are included in the article/**Supplementary Material**. Further inquiries can be directed to the corresponding author/s.

## References

[B1] Abu-Ria M. E. Elghareeb E. M. Shukry W. M. Abo-Hamed S. A. Ibraheem F. (2024). Mitigation of drought stress in maize and sorghum by humic acid: differential growth and physiological responses. BMC Plant Biol. 24, 514. doi: 10.1186/s12870-024-05184-4, PMID: 38849739 PMC11157776

[B2] Alsudays I. M. Alshammary F. H. Alabdallah N. M. Alatawi A. Alotaibi M. M. Alwutayd K. M. . (2024). Applications of humic and fulvic acid under saline soil conditions to improve growth and yield in barley. BMC Plant Biology 24, 191. doi: 10.1186/s12870-024-04863-6, PMID: 38486134 PMC10941484

[B3] Al-Taweel L. S. J. Al-Saadawi A. M. W. (2021). The effect of humic acid, vermicompost and nano-phosphorous on growth and yield characteristics of maize (Zea mays L.). IOP Conf. Ser: Earth Environ. Sci IOP Publish 923, 012080. doi: 10.1088/1755-1315/923/1/012080

[B4] Azeem K. Shah S. Ahmad N. Shah S. T. Khan F. Arafat Y. . (2015). Physiological indices, biomass and economic yield of maize influenced by humic acid and nitrogen levels. Russian Agric. Sci. 41, 115–119. doi: 10.3103/S1068367415020020

[B5] Baldi E. Toselli M. Marcolini G. Quartieri M. Cirillo E. Innocenti A. . (2010). Compost can successfully replace mineral fertilizers in the nutrient management of commercial peach orchard. Soil Use Manage. 26, 346–353. doi: 10.1111/j.1475-2743.2010.00286.x, PMID: 41834780

[B6] Bao S. D. (2000). Soil and Agro-Chemistry Analysis, third ed. (Beijing: China Agric Press).

[B7] Billah M. Khan M. Bano A. Hassan T. U. Munir A. Gurmani A. R. . (2019). Phosphorus and phosphate solubilizing bacteria: Keys for sustainable agriculture. Geomicrobiol. J. 36, 904–916. doi: 10.1080/01490451.2019.1654043, PMID: 41799851

[B8] Cozzolino V. Monda H. Savy D. Di Meo V. Vinci G. Smalla K. . (2021). Cooperation among phosphate-solubilizing bacteria, humic acids and arbuscular mycorrhizal fungi induces soil microbiome shifts and enhances plant nutrient uptake. Chem. Biol. Technol. Agric. 8, 31. doi: 10.1186/s40538-021-00230-x, PMID: 41853737

[B9] da Silva M. S. R. A. Tavares O. C. H. Ribeiro T. G. da Silva C. S. R. A. García-Mina J. M. . (2021). Humic acids enrich the plant microbiota with bacterial candidates for the suppression of pathogens. Appl. Soil Ecol. 168, 104146. doi: 10.1016/j.apsoil.2021.104146, PMID: 41853590

[B10] Dębska B. Długosz J. Piotrowska-Długosz A. Banach-Szott M. (2016). The impact of a bio-fertilizer on the soil organic matter status and carbon sequestration—results from a field-scale study. J. Soils Sediments 16, 2335–2343. doi: 10.1007/s11368-016-1430-5, PMID: 41853694

[B11] de Castro T. A. V. T. Berbara R. L. L. Tavares O. C. H. Mello D. F. d. G. Pereira E. G. de Souza C. d. C. B. . (2021). Humic acids induce a eustress state via photosynthesis and nitrogen metabolism leading to a root growth improvement in rice plants. Plant Physiol. Biochem. 162, 171–184. doi: 10.1016/j.plaphy.2021.02.043, PMID: 33684776

[B12] Dehsheikh A. B. Sourestani M. M. Zolfaghari M. Enayatizamir N. (2020). Changes in soil microbial activity, essential oil quantity, and quality of Thai basil as response to biofertilizers and humic acid. J. Clean. Prod. 256, 120439. doi: 10.1016/j.jclepro.2020.120439, PMID: 41853590

[B13] Elshamly A. M. S. Nassar S. M. A. (2023). Stimulating growth, root quality, and yield of carrots cultivated under full and limited irrigation levels by humic and potassium applications. Sci. Rep. 13, 14260. doi: 10.1038/s41598-023-41488-5, PMID: 37653028 PMC10471757

[B14] Elshayb O. M. Nada A. M. Sadek A. H. Ismail S. H. Shami A. Alharbi B. M. . (2022). The integrative effects of biochar and ZnO nanoparticles for enhancing rice productivity and water use efficiency under irrigation deficit conditions. Plants 11, 1416. doi: 10.3390/plants11111416, PMID: 35684189 PMC9183004

[B15] Feng F. X. Huang G. B. Chai Q. (2010). Tillage and straw management impacts on soil properties, root growth, and grain yield of winter wheat in Northwestern China. Crop Sci. 50, 1465–1473. doi: 10.2135/cropsci2008.10.0590

[B16] Guo Z. Wan S. Hua K. Yin Y. Chu H. Wang D. . (2020). Fertilization regime has a greater effect on soil microbial community structure than crop rotation and growth stage in an agroecosystem. Appl. Soil Ecol. 149, 103510. doi: 10.1016/j.apsoil.2020.103510, PMID: 41853590

[B17] Hartmann M. Six J. (2023). Soil structure and microbiome functions in agroecosystems. Nat. Rev. Earth Environ. 4, 4–18. doi: 10.1038/s43017-022-00366-w, PMID: 41844880

[B18] He H. Peng M. Lu W. Hou Z. Li J. (2022). Commercial organic fertilizer substitution increases wheat yield by improving soil quality. Sci. Total Environ. 851, 158132. doi: 10.1016/j.scitotenv.2022.158132, PMID: 36007638

[B19] Hopmans J. W. Qureshi A. S. Kisekka I. Munns R. Grattan S. R. Rengasamy P. . (2021). Critical knowledge gaps and research priorities in global soil salinity. Adv. Agron. 169, 1–191. doi: 10.1016/bs.agron.2021.03.001, PMID: 41853590

[B20] Ibrahim E. A. Ebrahim N. E. S. Mohamed G. Z. (2024). Mitigation of water stress in broccoli by soil application of humic acid. Sci. Rep. 14, 2765. doi: 10.1038/s41598-024-53012-4, PMID: 38307891 PMC10837118

[B21] Karlova R. Boer D. Hayes S. Testerink C. (2021). Root plasticity under abiotic stress. Plant Physiol. 187, 1057–1070. doi: 10.1093/plphys/kiab392, PMID: 34734279 PMC8566202

[B22] Kuzyakov Y. Gunina A. Zamanian K. Tian J. Luo Y. Xu X. . (2020). New approaches for evaluation of soil health, sensitivity and resistance to degradation. Frontiers of Agricultural Science and Engineering 7, 282–288. doi: 10.15302/J-FASE-2020338

[B23] Li S. Yao Y. Zhou C. Wang S. Liu Z. Liu Y. . (2025). Inorganic amendment can delay the degradation of organic amendment by enhancing its resistance and mitigating microbial activities in saline–alkali soils. Appl. Soil Ecol. 212, 106215. doi: 10.1016/j.apsoil.2025.106215, PMID: 41853590

[B24] Li Y. Fang F. Wei J. Wu X. Cui R. Li G. . (2019). Humic acid fertilizer improved soil properties and soil microbial diversity of continuous cropping peanut: A three-year experiment. Sci. Rep. 9, 12014. doi: 10.1038/s41598-019-48620-4, PMID: 31427666 PMC6700118

[B25] Li Z. Dou H. Zhang W. He Z. Li S. Xiang D. . (2023). The root system dominates the growth balance between the aboveground and belowground parts of cotton. Crop Environ. 2, 221–232. doi: 10.1016/j.crope.2023.09.001, PMID: 41853590

[B26] Ling J. Zhou J. Wu G. Zhao D. Q. Wang Z. T. Wen Y. . (2024). Deep-injected straw incorporation enhances subsoil quality and wheat productivity. Plant Soil 499, 207–220. doi: 10.1007/s11104-022-05660-6, PMID: 41853694

[B27] Liu M. Wang C. Wang F. Xie Y. (2019). Maize (Zea mays) growth and nutrient uptake following integrated improvement of vermicompost and humic acid fertilizer on coastal saline soil. Appl. Soil Ecol. 142, 147–154. doi: 10.1016/j.apsoil.2019.04.024, PMID: 41853590

[B28] Liu X. Yang J. Tao J. Yao R. Li W. Xie W. . (2023). Effects of the combined application of biochar and humic substances on the improvement of saline cropland in the Yellow River Delta of China. Land Degrad. Dev. 34, 4793–4809. doi: 10.1002/ldr.4810, PMID: 41848424

[B29] Luo G. Friman V. P. Chen H. Liu M. Wang M. Guo S. . (2018). Long-term fertilization regimes drive the abundance and composition of N-cycling-related prokaryotic groups via soil particle-size differentiation. Soil Biol. Biochem. 116, 213–223. doi: 10.1016/j.soilbio.2017.10.015, PMID: 41853590

[B30] Ma W. Ma L. Jiao J. Fahim A. M. Wu J. Tao X. . (2024). Impact of straw incorporation on the physicochemical profile and fungal ecology of saline–alkaline soil. Microorganisms 12, 277. doi: 10.3390/microorganisms12020277, PMID: 38399680 PMC10892582

[B31] Maji D. Misra P. Singh S. Kalra A. (2017). Humic acid rich vermicompost promotes plant growth by improving microbial community structure of soil as well as root nodulation and mycorrhizal colonization in the roots of Pisum sativum. Appl. Soil Ecol. 110, 97–108. doi: 10.1016/j.apsoil.2016.10.008, PMID: 41853590

[B32] Mao W. Kang S. Wan Y. Sun Y. Li X. Wang Y. (2016). Yellow River sediment as a soil amendment for amelioration of saline land in the Yellow River Delta. Land Degrad. Dev. 27, 1595–1602. doi: 10.1002/ldr.2323, PMID: 41848424

[B33] Maurel C. Nacry P. (2020). Root architecture and hydraulics converge for acclimation to changing water availability. Nat. Plants 6, 744–749. doi: 10.1038/s41477-020-0684-5, PMID: 32601421

[B34] Nallanthighal S. V. Enesi R. O. Thilakarathna M. S. Gorim L. Y. (2024). Agronomic responses and economic returns from wheat–canola rotation under Humalite and urea applications. Agron. J. 116, 3256–3272. doi: 10.1002/agj2.21681, PMID: 41848424

[B35] Nayab G. Zhou J. Jia R. Lv Y. Yang Y. Brown R. W. . (2022). Climate warming masks the negative effect of microplastics on plant-soil health in a silt loam soil. Geoderma 425, 116083. doi: 10.1016/j.geoderma.2022.116083, PMID: 41853590

[B36] Orlova N. Shakhnazarova V. Orlova E. Bityutskii N. Smirnova K. Ku S. . (2023). The taxonomic composition changes of bacteria and fungi in plant residue composts induced by biochar and calcium carbonate application. Agronomy 13, 2521. doi: 10.3390/agronomy13102521, PMID: 41725453

[B37] Ouni Y. Ghnaya T. Montemurro F. Abdelly C. Lakhdar A. (2014). The role of humic substances in mitigating the harmful effects of soil salinity and improve plant productivity. Int. J. Plant Prod. 8, 353–374.

[B38] Poirier V. Roumet C. Munson A. D. (2018). The root of the matter: Linking root traits and soil organic matter stabilization processes. Soil Biol. Biochem. 120, 246–259. doi: 10.1016/j.soilbio.2018.02.016, PMID: 41853590

[B39] Qiao L. Wang X. Smith P. Fan J. Lu Y. Emmett B. . (2022). Soil quality both increases crop production and improves resilience to climate change. Nat. Clim. Change 12, 574–580. doi: 10.1038/s41558-022-01376-8, PMID: 41844880

[B40] Saidimoradi D. Ghaderi N. Javadi T. (2019). Salinity stress mitigation by humic acid application in strawberry (Fragaria x ananassa Duch.). Scient Hortic. 256, 108594. doi: 10.1016/j.scienta.2019.108594, PMID: 41853590

[B41] Seyedbagheri M. M. (2010). Influence of humic products on soil health and potato production. Potato Res. 53, 341–349. doi: 10.1007/s11540-010-9177-7, PMID: 41853694

[B42] Shakoor A. Shakoor S. Rehman A. Ashraf F. Abdullah M. Shahzad S. M. . (2021). Effect of animal manure, crop type, climate zone, and soil attributes on greenhouse gas emissions from agricultural soils—A global meta-analysis. J. Clean. Prod. 278, 124019. doi: 10.1016/j.jclepro.2020.124019, PMID: 41853590

[B43] Shao G. Zhou J. Liu B. Alharbi S. A. Liu E. Kuzyakov Y. (2024). Carbon footprint of maize-wheat cropping system after 40-year fertilization. Sci. Total Environ. 926, 172082. doi: 10.1016/j.scitotenv.2024.172082, PMID: 38554958

[B44] Shrivastava P. Kumar R. (2014). Soil salinity: A serious environmental issue and plant growth promoting bacteria as one of the tools for its alleviation. Saudi J. Biol. Sci. 22, 123. doi: 10.1016/j.sjbs.2014.12.001, PMID: 25737642 PMC4336437

[B45] Song J. Guan X. Cui H. Liu L. Li Y. Li Y. . (2025). The impact of salt-tolerant plants on soil nutrients and microbial communities in soda saline-alkali lands of the Songnen plain. Front. Microbiol. 16, 1592834. doi: 10.3389/fmicb.2025.1592834, PMID: 40539100 PMC12177719

[B46] Sun X. Guo Y. Zeng L. Li X. Liu X. Li J. . (2021). Combined urea humate and wood vinegar treatment enhances wheat–maize rotation system yields and nitrogen utilization efficiency through improving the quality of saline–alkali soils. J. Soil Sci. Plant Nutr. 21, 1759–1770. doi: 10.1007/s42729-021-00477-1, PMID: 41853694

[B47] Wang C. L. Xu C. Y. Ma R. T. Li Q. R. Hu F. N. Zhao S. W. . (2025). Comparison for colloidal stability and aggregation behavior of fulvic and humic acids: effects of cations and pH. Front. Soil Sci. 5, 1452870. doi: 10.3389/fsoil.2025.1452870, PMID: 41853828

[B48] Wang R. Cao B. Sun Q. Song L. (2020). Response of grass interplanting on bacterial and fungal communities in a jujube orchard in Ningxia, northwest China. Heliyon 6, e03489. doi: 10.1016/j.heliyon.2020.e03489, PMID: 32154422 PMC7052399

[B49] Xu H. Wang S. Jiang N. Xie H. Chen Z. Zhang Y. . (2024). Organic fertilizer prepared by thermophilic aerobic fermentation technology enhanced soil humus and related soil enzyme activities. Soil Use Manage. 40(3), e13059. doi: 10.1111/sum.13059, PMID: 41834780

[B50] Yang F. Antonietti M. (2020). Artificial humic acids: sustainable materials against climate change. Adv Sci. 7, 1902992. doi: 10.1002/advs.201902992, PMID: 32154079 PMC7055563

[B51] Yang H. Cai Z. De Clerck C. Meersmans J. Colinet G. Zhang W. (2024). Long-term manuring enhanced compositional stability of glomalin-related soil proteins through arbuscular mycorrhizal fungi regulation. Agriculture 14, 1510. doi: 10.3390/agriculture14091510, PMID: 41725453

[B52] Zandonadi D. B. Matos C. R. R. Castro R. N. Spaccini R. Olivares F. L. Canellas L. P. . (2019). Alkamides: a new class of plant growth regulators linked to humic acid bioactivity. Chem. Biol. Technol. Agric. 6, 23. doi: 10.1186/s40538-019-0161-4, PMID: 41853737

[B53] Zhang X. Ding Z. Yang J. Cizmas L. Lichtfouse E. Sharma V. K. (2018). Efficient microwave degradation of humic acids in water using persulfate and activated carbon. Environ. Chem. Lett. 16, 1069–1075. doi: 10.1007/s10311-018-0721-z, PMID: 41853694

[B54] Zhang Z. Ma Y. Tian Y. Liu P. Zhang M. Liu Z. . (2024). Co-application of coated phosphate fertilizer and humic acid for wheat production and soil nutrient transport. Agronomy 14, 1621. doi: 10.3390/agronomy14081621, PMID: 41725453

