# Supplementary material for: Humic acid application improves soil quality and wheat yield in saline-alkali soils
Source: Front Plant Sci. 2026 Mar 24;17:1807251. doi: 10.3389/fpls.2026.1807251 (PMC13055627; doi:10.3389/fpls.2026.1807251)
Supplement: Supplementary file 1 [file SupplementaryFile1.docx]

**Text S1** Soil physicochemical analysis

The contents of water-soluble Na⁺, Ca²⁺, K^+^ and Mg²⁺ were determined by inductively coupled plasma mass spectrometry (ICP-MS) and atomic absorption spectrophotometry. Cl⁻ and SO₄²⁻ were measured using high-performance ion chromatography (HPIC). HCO₃⁻ was determined by neutralization titration. Soil EC and pH were measured using a conductivity meter and a digital pH meter, respectively, with a soil-to-water ratio of 1:5.

Alkali-hydrolyzable nitrogen (AHN), available phosphorus (AP), and available potassium (AK) were measured using the diffusion method, ammonium fluoride extraction method, and ammonium acetate extraction–flame photometry method, respectively. Soil organic matter (SOM) was determined by the potassium dichromate oxidation method. Total nitrogen (TN) was measured using the Kjeldahl digestion method. Total phosphorus (TP) was determined by the molybdenum antimony anti-spectrophotometry method. Total potassium (TK) was determined by flame photometry after digestion with hydrofluoric acid and perchloric acid.

The above determination methods of soil properties were based on the methods described by Bao (2000).

**Text S2** Sampling and analysis of wheat roots

Cleaned root samples were scanned using an Epson Perfect V750 Pro scanner (Epson Inc., Beijing, China), which had been fully submerged in deionized water beforehand. For the determination of root biomass (RB, g cm⁻³), the roots were subsequently dried in an 80°C oven to a constant weight. Root length (RL, cm cm⁻³), root surface area (RSA, cm² cm⁻³), and root volume (RV, cm³ cm⁻³) were calculated through analysis of root images using WinRHIZO software (Regent Instruments, Canada).

**Text S3** DNA extraction and high-throughput sequencing

Briefly, approximately 0.25 grams of rhizosphere soil sample was weighed out and used for DNA extraction with the OMEGA Soil DNA Kit (Cat. No. M5635-02; Omega Bio-Tek, Norcross, GA, USA). Samples were sequenced via Illumina® MiSeq (Genesky Biotechnologies Inc.). A total of 50,000 effective tags per sample were obtained for subsequent analysis.

The V4-V5 hypervariable regions of the 16S rRNA gene were amplified with the primers 515F (5'-GTGCCAGCMGCCGCGG-3') and 907R (5'-CCGTCAATTCMTTTRAGTTT-3'). The ITS1 hypervariable region of the ITS rRNA gene was amplified with the primers ITS1 (5'-CTTGGTCATTTAGAGGAAGTAA-3') and ITS2 (5'-GCTGCGTTCTTCATCGATGC-3').

Raw sequences were filtered, spliced, and chimera removed using QIIME (V1.8.0) software. High-quality sequences were clustered into OTUs at a similarity threshold of ≥ 97%, and the longest sequence within each cluster was selected as the representative sequence. Taxonomic annotation of OTUs was performed using the RDP-classifier database (Version 2.2; http://sourceforge.net/projects/rdp-classifier/). OTUs that failed the annotation were classified as “unidentified taxa”. Based on the OTU table, alpha diversity indices (Chao 1 and Shannon) of bacteria and fungi were calculated using the QIIME 2 microbiome analysis platform (https://qiime2.org).

**Text S4** Linear score of soil indicators

Soil indicators linear scoring using the following equations:

${SL}_{i}'=\frac{X}{X_{max}} (1)$

${SL}_{i}''=\frac{X_{min}}{X} (2)$

where “${SL}_{i}$”(including ${SL}_{i}'$ and ${SL}_{i}''$) is the linear score of the *i*^th^ indicator varying from 0 to 1, “*X*” denotes the measured value, “*X_max_*” and “*X_min_*” are the maximum and minimum mean values of the *i*^th^ indicator, respectively. Based on the sensitivity of soil quality, soil parameters were divided into two functions. A “more is better, ${SL}_{i}'$” scoring curve was used for each parameter where an increase results in an improvement in soil quality, (e.g., SOM, TP, AP), otherwise a “less is better, ${SL}_{i}''$” scoring curve was used (e.g., pH). In this study, except that C/N ratio was calculated using Eq. (2), other soil indicators was calculated using Eq. (1).

**References**

Bao SD (2000) Soil and Ago-Chemistry Analysis, 3rd ed. China Agric Press, Beijing

Vance ED, Brookes PC, Jenkinson DS (1987) An extraction method for measuring soil microbial biomass C. Soil Biol Biochem 19: 703-707. https://doi.org/10.1016/ 0038-0717(87)90052-6

**Table S1** Effect of humic acid (HA) on nitrogen content, phosphorus content, potassium content, and root traits of wheat plants. The HA treatments were CK, 0 Mg ha⁻¹, HA 3, 3 Mg ha⁻¹, HA 7.5, 7.5 Mg ha⁻¹, and HA 15, 15 Mg ha⁻¹.

| Treatments | Shoot N  （g cm^-2^） | Shoot P  （g cm^-2^） | Shoot K  （g cm^-2^） | Root biomass  (×10 g m^-1^) | Root length  (×10^3^ cm^3^ m^-1^) | Root surface area  (×10^2^ cm^2^ m^-1^) | Root volume  (×10 cm^3^ m^-1^) | Root N  （g cm^-2^） | Root P  （g cm^-2^） | Root K  （g cm^-2^） |
| --- | --- | --- | --- | --- | --- | --- | --- | --- | --- | --- |
| CK | 0.39±0.01b | 0.11±0.01b | 0.63±0.02b | 2.99±0.19b | 7.95±0.62b | 4.33±0.23b | 3.41±0.12b | 0.18±0.01c | 0.08±0.01c | 0.25±0.02b |
| HA 3 | 0.42±0.01ab | 0.12±0.01a | 0.75±0.03a | 3.49±0.23b | 9.48±0.72a | 5.03±0.45ab | 4.01±0.23a | 0.21±0.01b | 0.08±0.01bc | 0.31±0.02a |
| HA 7.5 | 0.47±0.01a | 0.14±0.01a | 0.86±0.02a | 4.15±0.19a | 10.22±0.35a | 5.51±0.54a | 4.24±0.09a | 0.23±0.01a | 0.10±0.01ab | 0.33±0.02a |
| HA 15 | 0.48±0.01a | 0.14±0.01a | 0.90±0.05a | 4.19±0.34a | 10.48±0.41a | 5.64±0.31a | 4.27±0.10a | 0.24±0.01a | 0.10±0.01a | 0.34±0.02a |
| One-way ANOVA | |  |  |  |  |  |  |  |  |  |
| Treatment | 0.016 | 0.001 | **<0.001** | **<0.001** | 0.002 | **<0.001** | **<0.001** | **<0.001** | 0.003 | 0.005 |

Different letters denote significant differences among treatments at 0.05 level. Values are means±standard deviations (*n* = 3)

**Table S2** Effect of humic acid (HA) on soil eight major ions, pH, EC. The HA treatments were CK, 0 Mg ha⁻¹, HA 3, 3 Mg ha⁻¹, HA 7.5, 7.5 Mg ha⁻¹, and HA 15, 15 Mg ha⁻¹.

| Treatments | pH | EC | Cl^-^  (g kg-1) | K^+^  (g kg^-1^) | SO_4_^2-^  (g kg^-1^) | Na^+^  (g kg^-1^) | HCO_3_^-^  (g kg^-1^) | Ca^2+^  (g kg^-1^) | Mg^2+^  (g kg^-1^) |
| --- | --- | --- | --- | --- | --- | --- | --- | --- | --- |
| 0-20 cm |  |  |  |  |  |  |  |  |  |
| CK | 8.14±0.03a | 1.40±0.15a | 1.06±0.18a | 0.07±0.03a | 1.11±0.06a | 0.30±0.03a | 0.23±0.03a | 0.39±0.07a | 0.24±0.03a |
| HA 3 | 8.04±0.01b | 1.28±0.08ab | 1.03±0.12a | 0.08±0.05a | 1.19±0.12a | 0.20±0.07b | 0.21±0.01a | 0.32±0.05ab | 0.22±0.03ab |
| HA 7.5 | 8.00±0.02b | 1.02±0.07b | 0.58±0.12b | 0.07±0.02a | 0.86±0.05b | 0.17±0.02b | 0.21±0.02a | 0.25±0.05b | 0.15±0.01ab |
| HA 15 | 7.98±0.03b | 1.06±0.10b | 0.68±0.05b | 0.06±0.05a | 0.86±0.10b | 0.17±0.06b | 0.21±0.02a | 0.25±0.02b | 0.17±0.07b |
| 20-40 cm |  |  |  |  |  |  |  |  |  |
| CK | 8.26±0.07a | 1.07±0.10a | 0.70±0.17a | 0.01±0.01a | 1.10±0.12a | 0.42±0.10a | 0.23±0.01a | 0.19±0.06a | 0.18±0.07a |
| HA 3 | 8.16±0.07a | 1.01±0.05a | 0.86±0.11ab | 0.01±0.01a | 1.02±0.12a | 0.27±0.04b | 0.23±0.01a | 0.30±0.13a | 0.21±0.09a |
| HA 7.5 | 8.13±0.09a | 0.79±0.09b | 0.49±0.09b | 0.01±0.01a | 1.02±0.13a | 0.19±0.03b | 0.22±0.02a | 0.16±0.06a | 0.16±0.08a |
| HA 15 | 8.09±0.08a | 0.95±0.06ab | 0.55±0.20b | 0.01±0.01a | 1.02±0.13a | 0.20±0.07b | 0.21±0.03a | 0.17±0.03a | 0.15±0.03a |
| Two-way ANOVA | |  |  |  |  |  |  |  |  |
| Treatment (T) | **<0.001** | **<0.001** | **<0.001** | 0.910 | 0.674 | 0.063 | 0.346 | **<0.001** | 0.307 |
| Layer (L) | **<0.001** | **<0.001** | **<0.001** | **<0.001** | 0.802 | 0.131 | 0.274 | 0.029 | 0.466 |
| T×L | 0.991 | 0.238 | 0.339 | 0.899 | 0.804 | 0.778 | 0.923 | 0.445 | 0.820 |

Different letters denote significant differences among treatments at 0.05 level. Values are means ± standard deviations (*n* = 3)

**Table S3** Effect of humic acid (HA) on soil available phosphorus (AP), available potassium (AK), alkaline hydrolyzable nitrogen (AHN), soil organic matter (SOM), total nitrogen (TN) and total phosphorus (TP). The HA treatments were CK, 0 Mg ha⁻¹, HA 3, 3 Mg ha⁻¹, HA 7.5, 7.5 Mg ha⁻¹, and HA 15, 15 Mg ha⁻¹.

| Treatments | AP  (mg kg^-1^) | AK  (mg kg^-1^) | AHN  (mg kg^-1^) | SOM  (g kg^-1^) | TN  (g kg^-1^) | TP  (g kg^-1^) |
| --- | --- | --- | --- | --- | --- | --- |
| 0-20 cm |  |  |  |  |  |  |
| CK | 12.98±1.53b | 118.38±6.31b | 56.22±4.51b | 1.14±0.05b | 0.07±0.01ab | 0.88±0.04b |
| HA 3 | 14.88±0.73b | 121.53±3.95b | 67.51±2.75ab | 1.29±0.06ab | 0.07±0.01b | 0.95±0.02ab |
| HA 7.5 | 18.31±0.92a | 145.81±4.41a | 70.21±4.93a | 1.33±0.07a | 0.08±0.01ab | 0.96±0.04a |
| HA 15 | 18.48±0.73a | 143.99±4.05a | 69.96±4.84a | 1.34±0.09a | 0.08±0.01a | 0.92±0.02a |
| 20-40 cm |  |  |  |  |  |  |
| CK | 2.88±0.23b | 58.49±8.54b | 25.76±3.59a | 0.42±0.02b | 0.03±0.01a | 0.53±0.05b |
| HA 3 | 3.41±0.29b | 70.24±7.40ab | 28.02±6.41a | 0.44±0.03ab | 0.03±0.01a | 0.61±0.04ab |
| HA 7.5 | 4.25±0.18a | 81.46±7.28a | 30.73±5.13a | 0.48±0.01ab | 0.03±0.01a | 0.65±0.04a |
| HA 15 | 4.39±0.25a | 84.99±7.82a | 23.95±5.48a | 0.50±0.03a | 0.04±0.01a | 0.67±0.05a |
| Two-way ANOVA | |  |  |  |  |  |
| Treatment (T) | **<0.001** | **<0.001** | **<0.001** | **<0.001** | **<0.001** | **<0.001** |
| Layer (L) | **<0.001** | **<0.001** | **<0.001** | **<0.001** | **<0.001** | **<0.001** |
| T×L | **<0.001** | 0.393 | 0.086 | 0.129 | 0.906 | 0.789 |

Different letters denote significant differences among treatments at 0.05 level. Values are means ± standard deviations (n = 3)

**Table S4** Effect of humic acid (HA) on the α-diversity index of soil bacterial and fungal communities. The HA treatments were CK, 0 Mg ha⁻¹, HA 3, 3 Mg ha⁻¹, HA 7.5, 7.5 Mg ha⁻¹, and HA 15, 15 Mg ha⁻¹.

| Treatments | Bacteria | | Fungi | |
| --- | --- | --- | --- | --- |
|  | Chao1 | Shannon | Chao1 | Shannon |
| CK | 3346.32±247.82a | 10.67±0.15a | 371.72±67.13a | 3.66±0.56b |
| HA3 | 3122.03±459.66a | 10.53±0.21a | 264.70±72.86b | 4.02±0.57ab |
| HA7.5 | 3242.51±469.06a | 10.58±0.19a | 319.01±77.64ab | 4.37±0.66a |
| HA15 | 3282.39±422.15a | 10.55±0.25a | 304.55±72.01b | 4.33±0.59a |
| One-way ANOVA | | |  |  |
| Treatment (T) | 0.664 | 0.463 | 0.019 | 0.039 |

Different letters denote significant differences among treatments at 0.05 level. Values are means ± standard deviations (*n*=3).


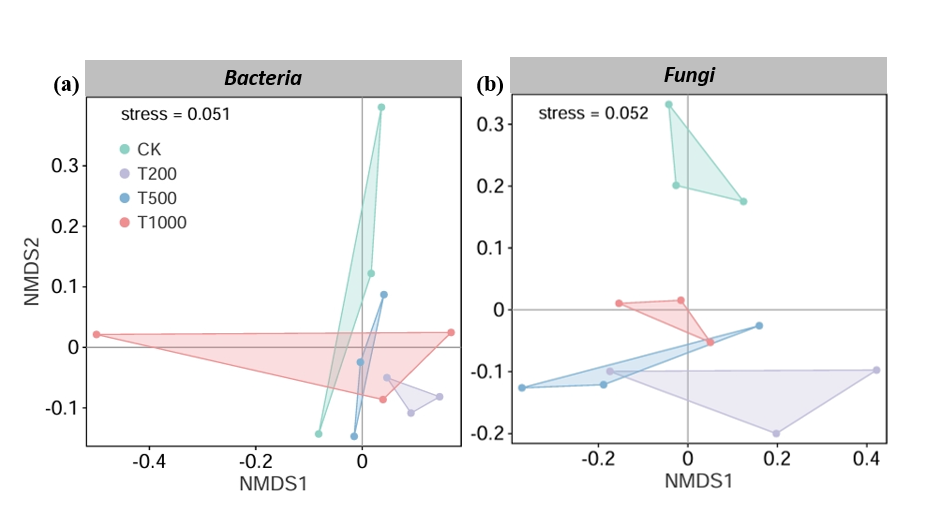


**Fig. S1** Nonmetric multidimensional scaling (NMDS) analysis of bacterial (a) and fungal (b) communities in rhizosphere soil based on the Bray-Curtis distance matrix. The HA treatments were CK, 0 Mg ha⁻¹, HA 3, 3 Mg ha⁻¹, HA 7.5, 7.5 Mg ha⁻¹, and HA 15, 15 Mg ha⁻¹.

**
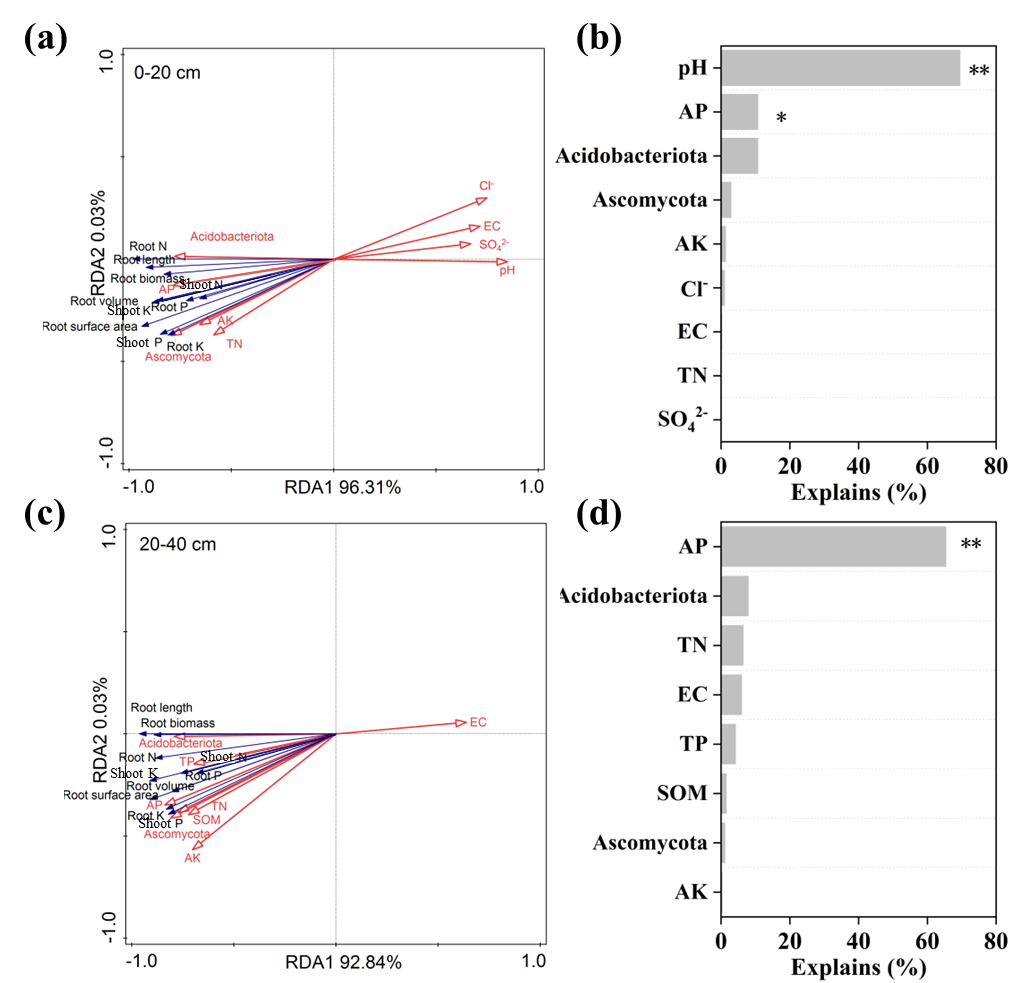
**

**Fig. S2** Redundancy analysis (RDA) illustrating the relationships between soil physicochemical properties, key microbial taxa, and plant nutrient uptake in plant and root. Shoot P: plant phosphorus content; Shoot N: Plant Nitrogen Content; Shoot K: Plant Potassium Content; Root N: root nitrogen content; Root P: root phosphorus content; Root biomass: root weight; Root length: root length; Root volume: root surface area; AK: available potassium; Cl⁻: chloride ion; SO₄²⁻: sulfate ion; AP: available phosphorus; TN: total nitrogen; EC: electrical conductivity; pH: potential of hydrogen; SOM: soil organic matter; Acidobacteriota: Acidobacteria; Ascomycota: Ascomycetes.
